# Nonhuman primate to human immunobridging to infer the protective effect of an Ebola virus vaccine candidate

**DOI:** 10.1038/s41541-020-00261-9

**Published:** 2020-12-17

**Authors:** Ramon Roozendaal, Jenny Hendriks, Thierry van Effelterre, Bart Spiessens, Liesbeth Dekking, Laura Solforosi, Dominika Czapska-Casey, Viki Bockstal, Jeroen Stoop, Daniel Splinter, Sarah Janssen, Ben van Baelen, Nadia Verbruggen, Jan Serroyen, Eline Dekeyster, Ariane Volkmann, Yvonne Wollmann, Ricardo Carrion, Luis D. Giavedoni, Cynthia Robinson, Maarten Leyssen, Macaya Douoguih, Kerstin Luhn, Maria Grazia Pau, Jerry Sadoff, An Vandebosch, Hanneke Schuitemaker, Roland Zahn, Benoit Callendret

**Affiliations:** 1 Janssen Vaccines & Prevention B.V., Leiden, The Netherlands; 2 Janssen R&D, Beerse, Belgium; 3 Bavarian Nordic, Munich, Germany; 4 Texas Biomedical Research Institute, San Antonio, TX USA; 5 Present Address: argenx BV, Gent, Belgium; 6 Present Address: BVB Clin Consult BVBA, Mechelen, Belgium; 7 Present Address: Galapagos NV, Mechelen, Belgium; 8 Present Address: FGK Clinical Research, Munich, Germany

**Keywords:** Immunology, Vaccines

## Abstract

It has been proven challenging to conduct traditional efficacy trials for Ebola virus (EBOV) vaccines. In the absence of efficacy data, immunobridging is an approach to infer the likelihood of a vaccine protective effect, by translating vaccine immunogenicity in humans to a protective effect, using the relationship between vaccine immunogenicity and the desired outcome in a suitable animal model. We here propose to infer the protective effect of the Ad26.ZEBOV, MVA-BN-Filo vaccine regimen with an 8-week interval in humans by immunobridging. Immunogenicity and protective efficacy data were obtained for Ad26.ZEBOV and MVA-BN-Filo vaccine regimens using a fully lethal EBOV Kikwit challenge model in cynomolgus monkeys (nonhuman primates [NHP]). The association between EBOV neutralizing antibodies, glycoprotein (GP)-binding antibodies, and GP-reactive T cells and survival in NHP was assessed by logistic regression analysis. Binding antibodies against the EBOV surface GP were identified as the immune parameter with the strongest correlation to survival post EBOV challenge, and used to infer the predicted protective effect of the vaccine in humans using published data from phase I studies. The human vaccine-elicited EBOV GP-binding antibody levels are in a range associated with significant protection against mortality in NHP. Based on this immunobridging analysis, the EBOV GP-specific-binding antibody levels elicited by the Ad26.ZEBOV, MVA-BN-Filo vaccine regimen in humans will likely provide protection against EBOV disease.

## Introduction

The frequency and magnitude of Ebola virus (EBOV) outbreaks are apparently on the increase^1^, emphasizing the need for prophylactic vaccines^2^, as well as reactive vaccination and other measures to rapidly contain outbreaks. However, conducting traditional randomized controlled efficacy studies of a prophylactic Ebola vaccine is only feasible in large outbreaks and brings logistical challenges.

Anticipating circumstances under which vaccine efficacy demonstration may not be technically or ethically feasible, guidelines for demonstrating a likelihood of clinical benefit include: the FDA Animal Rule^3^, European Medicine Agency conditional approval^4^ or approval under exceptional circumstances^5^, and Health Canada extraordinary use of a new drug^6^. These guidelines stipulate that an immunological marker that correlates with protection in a suitable animal model could be used to demonstrate likelihood of clinical benefit as a basis for licensure, with additional postlicensure commitments. Recently, BioThrax^®^ became the first vaccine licensed under the FDA Animal Rule^7^. While immunobridging assumes that the protective mechanism is conserved between the animal model and humans, the immunological marker selected for immunobridging only needs to correlate with the desired benefit, and is not necessarily involved in the mechanism of protection^3^.

**Fig. 1:**
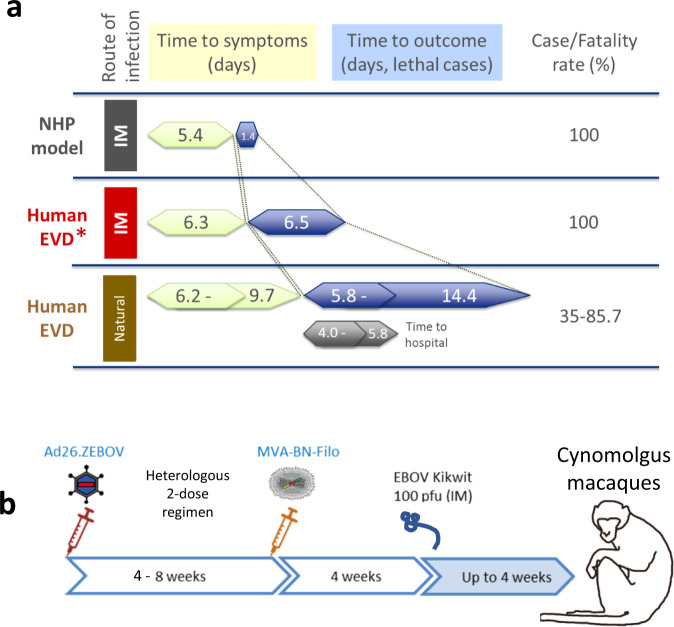
Experiment schematic and comparison of Ebola virus disease course in NHP and humans. **a** The disease course and outcome of EVD was compared between untreated NHP and humans*. As far as the authors are aware, IM infection in humans via needle reusage is only identified in a single report^53^. There is rapid disease progression in NHP relative to humans, irrespective of route of infection. Duration indicated for NHP represents the average of 13 untreated controls from seven independent NHP studies (C25#1, 12, C29#1, C29#2, C29#8, TO14#1, TO14#2). In humans, time to symptoms, time to outcome, and time to hospital are based on cited papers^11–19^, and shown as the range of the mean values reported in the cited studies. Symptom onset in NHP was defined based on recorded clinical score. **b** Experimental schematic of immunogenicity and efficacy studies in NHP. Vaccine regimens containing Ad26.ZEBOV and MVA-BN-Filo were tested with variations in dose, dose interval, dose order, and valency of the adenovector vaccine. Four weeks after the final vaccination, NHP were challenged with EBOV Kikwit (IM) at a target dose of 100 pfu (actual range 50–1615 pfu), and followed up for signs and symptoms of EVD for up to 4 weeks. EBOV Ebola virus, EVD Ebola virus disease, IM intramuscular, NHP nonhuman primates.

For EBOV disease (EVD), nonhuman primates (NHP) are the most relevant animal model^8,9^, exhibiting the major hallmarks of hemorrhagic fever such as clotting abnormalities as well as liver and kidney damage, albeit with differences in disease course and severity associated with a higher lethality rate^10^. In this case, cynomolgus monkeys (*Macaca fascicularis*) are used, in which EVD appears stringent, even when compared to the most severe human cases (Fig. 1a, see legend for references). In humans, the mean reported time from infection to death of EVD ranges from 12 days to well over 3 weeks, whereas NHP succumb to disease within 7 days following intramuscular (IM) challenge with 100 plaque forming units (pfu) of EBOV Kikwit. By the time symptomatic patients with EVD arrive in a hospital, EBOV challenged NHP have already succumbed to disease, due to the particularly rapid disease progression in NHP (mean survival 1.4 days after symptom onset) relative to humans (time to death after symptom onset 5.8‒14.4 days for lethal cases [range of means])^11–19^. The high level of genetic homology between NHP and humans and their comparative immunology have made NHP the animal model of choice for studies of vaccine immunogenicity^20,21^. Thus, while the NHP model is considered a suitable disease model for vaccine testing^22^, there is no straightforward translation of the inferred protective effect in humans to human efficacy.

In the current work, we identify the concentration of EBOV surface glycoprotein (GP)-binding antibodies elicited by a heterologous two-dose vaccine regimen with Ad26.ZEBOV and MVA-BN-Filo as a strong predictor of survival after IM challenge of cynomolgus monkeys (*Macaca fascicularis*) with EBOV. Vaccine-elicited GP-binding antibody levels were also associated with attenuated disease, delayed progression, and a decrease in viral load, providing additional indications of a protective effect. The same vaccine regimen was used in phase I studies, where it also elicited strong EBOV GP-binding antibody responses detected by the same enzyme-linked immunosorbent assay (ELISA)^23–26^. Immunobridging based on EBOV GP-binding antibodies is used to infer the protective effect of the Ad26.ZEBOV, MVA-BN-Filo vaccine regimen in humans.

## Results

### Protective efficacy in NHP

IM challenge of NHP was performed at a target dose of 100 pfu of EBOV Kikwit. It was observed that a dose of 0.5 pfu was lethal in 10/10 untreated NHP (Supplementary Fig. 1), indicating that a dose of 100 pfu represents at least a 200-fold lethal dose. Challenge with 100 pfu in nonvaccinated NHP, uniformly resulted in first signs of EVD at day 5 or 6 and a lethal outcome, on average, 1.4 days later (*n* = 13, Fig. 1a). While the actual dose of infectious virus based on back titration varied from 50–1615 pfu, this did not result in apparent differences in the disease course.

**Fig. 2:**
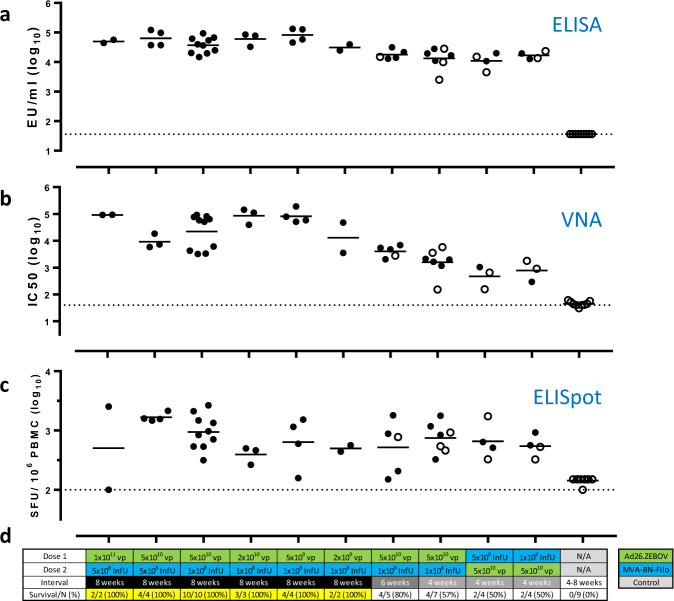
Immunogenicity and protective efficacy of Ad26.ZEBOV, MVA-BN-Filo regimens in NHP. Nonsurviving NHP are depicted by open circles. **a** EBOV GP-binding antibody concentrations (ELISA units [EU]/mL, log_10_ transformed) by regimen as determined by Filovirus Animal Non-Clinical Group (FANG) EBOV GP ELISA (BBRC); group means are indicated; samples below the lower limit of detection (LOD) are plotted at a common value (dotted line). **b** EBOV neutralizing antibody titers (IC_50_, log_10_ transformed) as determined by pseudovirus neutralization assay (psVNA; Monogram); group means are indicated; dotted line is LOD. Three samples could not be analyzed due to sample volume limitations. **c** EBOV GP-reactive IFN-γ T cells (spot-forming units [SFU]/10^6^ cells, log_10_ transformed) as enumerated by IFN-γ ELISpot (TBRI); group means are indicated; samples below LOD are plotted at a common value (dotted line). **d** Table shows vaccine dose, dose order, and dose interval, as well as the associated survival after challenge. Additional regimens tested, as well as an overview by regimen can be found in Supplementary Fig. 2 and Supplementary Table 1. Dose of Ad26.ZEBOV (green) and MVA-BN-Filo (blue) as well as dose interval (shades of red and pink). Yellow shading identifies regimens with 100% protection. ELISA enzyme-linked immunosorbent assay, IFN-γ ELISpot interferon-gamma enzyme-linked immunospot, EU ELISA units, IC_50_ half-maximal inhibitory concentration, InfU infectious units, LOD limit of detection, psVNA pseudovirus neutralization assay, SFU spot-forming units, PBMC peripheral blood mononuclear cells, vp viral particles.

All tested vaccine regimens were immunogenic, eliciting EBOV GP-binding antibodies, EBOV neutralizing antibodies, and EBOV GP-reactive T cells (Fig. 2a–c). The highest protective efficacy (100%) was obtained with Ad26.ZEBOV, MVA-BN-Filo regimens with an 8-week interval between doses. Immunization with Ad26.ZEBOV concentrations of 2 × 10^9^ —1 × 10^11^ viral particles (vp) followed 8 weeks later by MVA-BN-Filo (1 × 10^8^ infectious units [InfU]) provided protection in 25/25 NHP (Fig. 2d). Shorter intervals between vaccinations were associated with partial protection. Ad26.ZEBOV and MVA-BN-Filo immunization (5 × 10^10^ vp and 1 × 10^8^ InfU, respectively) with 6- or 4-week intervals resulted in protection of 4/5 NHP and 4/7 NHP, respectively. Regimens where MVA-BN-Filo instead of Ad26.ZEBOV was administered as the first dose also resulted in partial protective efficacy. MVA-BN-Filo (5 × 10^8^ InfU) followed by Ad26.Filo (1.2 × 10^11^ vp) 8 weeks later resulted in protection of 5/6 NHP, whereas MVA-BN-Filo (1–5 × 10^8^ InfU) followed by Ad26.ZEBOV (5 × 10^10^ vp) or Ad26.Filo (1.2 × 10^11^ vp) 4 weeks later gave protection in 4/8 and 1/4 NHP, respectively (Fig. 2d). Results for additional vaccine regimens are shown in Supplementary Fig. 2. These regimens contain a trivalent filovirus vaccine Ad26.Filo, which comprises Ad26.ZEBOV as a 1:1:1 mixture with Ad26 vectors expressing MARV and SUDV GPs. All vaccine regimens were immunogenic and had varying degrees of efficacy. Primary efficacy data for six NHP shown in Supplementary Fig. 2 were already reported elsewhere^27^.

In all studies, blood was sampled 1 week prior to challenge for analysis of humoral and cellular immune responses. The Ad26.ZEBOV, MVA-BN-Filo 2-dose regimen with an 8-week interval between doses, which conferred full protection (Fig. 2d), was associated with the highest levels of EBOV GP-binding (Fig. 2a) and neutralizing (Fig. 2b) antibodies. Based on GP-reactive T cells, as measured by interferon-gamma enzyme-linked immunospot (IFN-γ ELISpot), no clear separation between survivors and nonsurvivors was observed (Fig. 2c). Similar observations were made for additional vaccine regimens tested (Supplementary Fig. 2).

### Immunological correlates of Ad26.ZEBOV, MVA-BN-Filo protection in NHP

The 100% survival observed in these studies for the Ad26.ZEBOV, MVA-BN-Filo regimen with an 8-week interval between doses precluded conducting specific correlate analysis for this vaccine regimen. Therefore, logistic regression models were based on data from all vaccinated NHP for all vaccine regimens combined (Supplementary Table 1). Logistic regression analysis describes the probability of the study outcome (survival) as a function of humoral or cellular measures of vaccine immunogenicity.

**Fig. 3:**
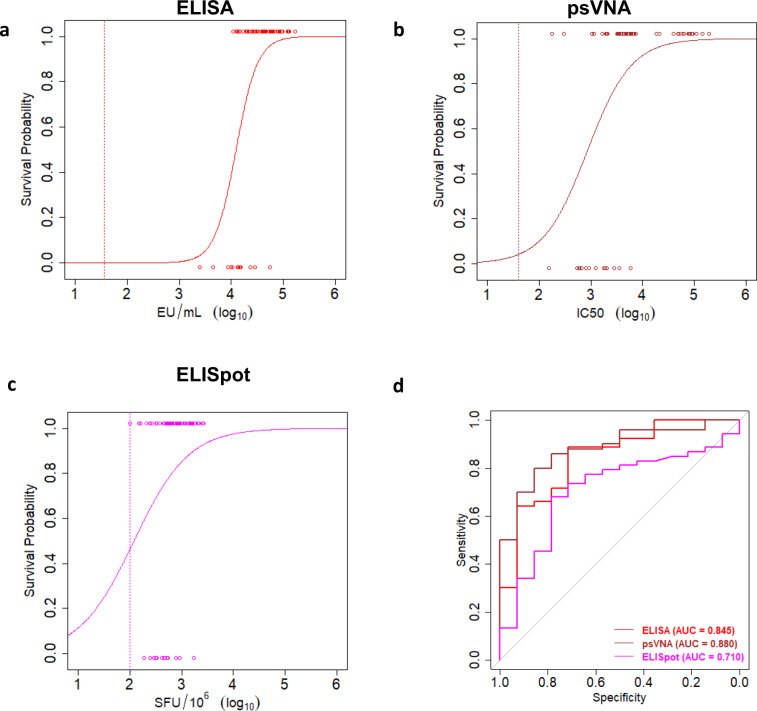
Immunological correlates of protection in NHP. Graphs represent logistic regression models with immune parameters as predictors of survival after EBOV Kikwit challenge (100 pfu, IM) in cynomolgus monkeys for: **a** EBOV GP-binding antibody levels (ELISA, red), and **b** EBOV neutralizing antibodies (psVNA, brown). **c** EBOV GP-reactive T cells (IFN-γ ELISpot, purple). Individual immune response levels are identified with open circles and the associated survival as a binary variable with survival as 1 (top) and nonsurvival as 0 (bottom). Assay LODs are indicated by dotted lines. **d** ROC curves for sensitivity and specificity of GP-binding antibody levels (red), EBOV neutralizing antibodies (brown) and EBOV GP-reactive T cells (purple) in predicting NHP survival after challenge. ROC AUC are indicated in the panel. AUC area under the curve, ELISA enzyme-linked immunosorbent assay, IFN-γ ELISpot interferon-gamma enzyme-linked immunospot, EU ELISA units, IC_50_ half-maximal inhibitory concentration, LOD limit of detection, psVNA pseudovirus neutralization assay, SFU spot-forming units.

Survival was used as a binary outcome (0 = death; 1 = survival) with a single immunological parameter as a covariate. A significant positive slope for the logistic model then indicates that increasing levels of immunogenicity are positively correlated to survival. GP-binding antibody levels (Fig. 3a, Filovirus Animal Non-Clinical Group (FANG) EBOV GP ELISA) (*n* = 67), EBOV neutralizing antibody levels (Fig. 3b, psVNA) (*n* = 64), and GP-reactive T-cell responses (Fig. 3c, IFN-γ ELISpot) (*n* = 67) were each significantly correlated to challenge outcome (slopes, *p* < 0.0001 for ELISA and psVNA, *p* = 0.045 for IFN-γ ELISpot) (Fig. 3a–c). For all parameters, there was some overlap between the values associated with survival and lethal outcome.

The logistic model based on T cells showed a lower discriminatory capacity than the model based on GP-binding antibodies (area under the curve [AUC] of the receiver operating characteristic [ROC] curve of 0.710 and 0.845, respectively) (Fig. 3d), in line with a large interindividual variation in GP-reactive T cells per regimen. GP-binding (Fig. 3a) and GP-neutralizing antibody levels (Fig. 3b) showed a better separation between the levels associated with survival and lethal outcome, similar sensitivity and specificity based on the ROC AUC (Fig. 3d). Since GP-binding and GP-neutralizing antibodies showed similar associations with protection, and GP-binding antibodies are measured in a more robust assay with less assay variation, GP-binding antibodies were selected for immunobridging. Survival probability as a function of GP-binding antibody levels in NHP increases steeply from 3.5 to 4.5 log_10_ EU/mL, though a target threshold for protection could not be identified.

### Refinement of GP-binding antibody logistic model by vaccine dose-down studies

After selection of GP-binding antibodies for immunobridging based on retrospective analysis, it was prospectively decided to conduct two additional challenge studies to finalize the logistic model for immunobridging. These studies tested lower (i.e., suboptimal) vaccine doses to characterize the correlation between GP-binding antibody responses and survival across a wider range of GP-binding antibody concentrations, and in particular at lower levels. Data from these two additional studies were used to refine the logistic model based on GP-binding antibody concentrations and survival.

**Fig. 4:**
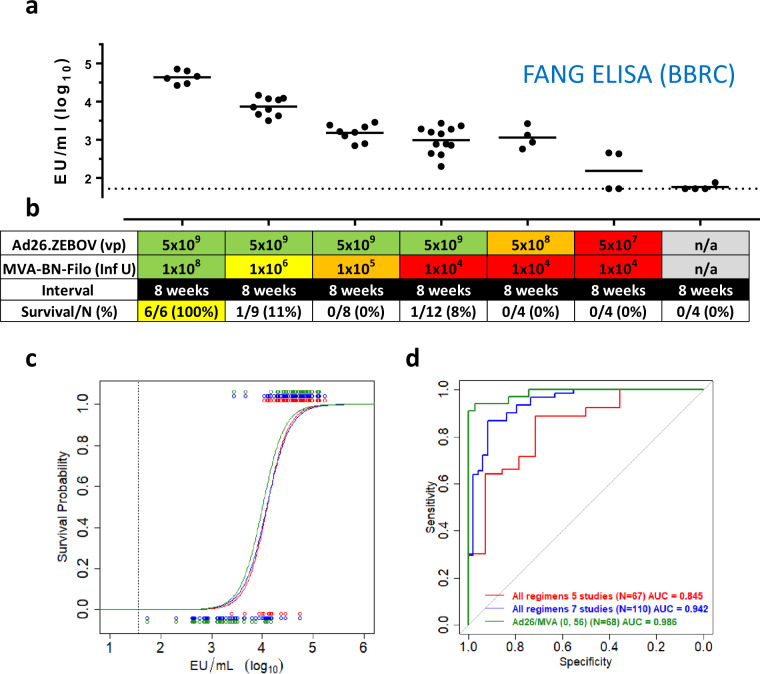
Vaccine dose-down studies refine logistic model based on GP-binding antibody levels for immunobridging. **a** EBOV GP-binding antibody concentrations (EU/mL, log_10_ transformed) as determined by FANG ELISA (BBRC); group means are indicated; dotted line is LOD. **b** Table identifies dose of Ad26.ZEBOV and MVA-BN-Filo with highest dose represented in green and lowest dose in red. Vaccine dose interval is 8 weeks. An overview by regimen can be found in Supplementary Table 4. **c** Logistic regression models of GP-binding antibodies as predictor of survival after EBOV Kikwit challenge (100 pfu, IM) in NHP for all vaccine regimens combined from five NHP studies (red), all regimens combined from seven NHP studies (blue), Ad26.ZEBOV, MVA-BN-Filo regimen with an 8-week interval only (green). Individual immune response levels are identified with open circles and the associated survival as a binary variable with survival as 1 (top) and nonsurvival as 0 (bottom). Assay LOD is indicated by dotted line. **d** ROC curves for sensitivity and specificity of GP-binding antibody levels in predicting NHP survival after challenge for all vaccine regimens combined from five NHP studies (red), all regimens combined from seven NHP studies (blue), Ad26.ZEBOV, MVA-BN-Filo regimen with an 8-week interval only (green). ROC AUC are indicated in the panel. BBRC Battelle Biomedical Research Institute, ELISA enzyme-linked immunosorbent assay, EU ELISA units, FANG Filovirus Animal Non-Clinical Group, InfU infectious units, vp viral particles.

As expected, GP-binding antibody responses decreased with decreasing vaccine doses (Fig. 4a). Ad26.ZEBOV (5 × 10^9^ vp) followed by MVA-BN-Filo immunization (1 × 10^8^ InfU) 8 weeks later elicited fully protective immunity against EBOV challenges in both studies, consistent with previous results (Fig. 2). In addition, GP-binding antibody levels were strongly correlated with EBOV neutralizing antibodies (shown in Supplementary Fig. 3 for Ad26.ZEBOV, MVA-BN-Filo regimen with an 8-week interval between doses, Pearson correlation 0.94).

Ad26.ZEBOV (5 × 10^9^ vp) combined with 1 × 10^6^, 1 × 10^5^, or 1 × 10^4^ InfU MVA-BN-Filo provided protection in 1/9, 0/8, and 1/12 NHP, respectively (Fig. 4b). Regimens composed of either 5 × 10^8^ vp or 5 × 10^7^ vp Ad26.ZEBOV, in combination with 1 × 10^4^ InfU MVA-BN-Filo, did not provide protection (0/4 NHP each).

While the updated logistic model based on all studies combined (Fig. 4c, blue line, *n* = 110) is nearly indistinguishable from the model built based on the first five NHP studies (red line, *n* = 67), the specificity and sensitivity for predicting outcome were markedly improved (Fig. 4d), from ROC AUC of 0.845 to 0.942. Thus, the observed relationship between vaccine-elicited GP-binding antibodies and survival outcome was confirmed in the prospective studies, with improved discrimination between survivors and nonsurvivors. Challenge breakthrough data from the groups that received the lower doses of Ad26.ZEBOV and MVA-BN-Filo with an 8-week interval also for the first time permitted the construction of a logistic model based on a single regimen (*n* = 68), rather than all regimens combined (*n* = 110); for instance, excluding data from regimens with different dose sequence and dose interval. The logistic model for the single-regimen Ad26.ZEBOV and MVA-BN-Filo with an 8-week interval, had the highest sensitivity and specificity (ROC AUC = 0.986) (Fig. 4d). This logistic model also had the highest discriminatory capacity, with a coefficient of discrimination (CoD) of 0.78.

We hypothesized that the protection variation against lethal challenge outcomes was solely due to differences in immunogenicity of different vaccination regimens. It was statistically evaluated whether dose sequence, dose interval, and vaccine valency contributed to the discriminatory capacity of the logistic model, in addition to GP-binding antibody level. The purpose of these analyses was to assess whether any of these additional covariates contribute additional discriminatory capacity relative to the model based on GP-binding antibody concentration alone. To verify this, we computed the adjusted CoD, which is the difference between the mean predicted survival probabilities in survivors and nonsurvivors for the logistic model with GP-binding antibodies with and without each of these variables adjusted for the number of covariates. In addition, the percent prediction explained (PPE)^7^ was calculated for the model based on GP-binding antibody levels versus the models with GP-binding antibody levels and an additional variable, in analogy to Fay et al.^7^. None of the tested covariates led to a marked change in the adjusted CoD, and the PPE estimates are all close to 100, indicating that the impact of a vaccine regimen is predominantly realized through GP-binding antibody levels, and dose sequence, dose interval, and valency do not contribute significant additional discriminatory capacity (Supplementary Table 2). In addition, vaccine dose was not an independent predictor of outcome for the logistic model based on the Ad26.ZEBOV, MVA-BN-Filo regimen (Supplementary Table 3), confirming that the main effect is related to the level of GP-binding antibodies, and that the model based on GP-binding antibodies alone can be used as an indicator of the protective effect of the vaccine regimen in humans.

### Inferring vaccine protective effect based on GP-binding antibody levels from phase I studies

**Fig. 5:**
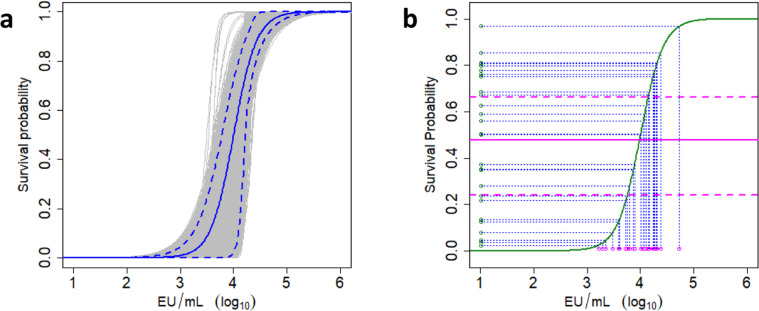
Immunobridging of human EBOV GP-binding antibodies to the NHP-challenge model. **a** Visual representation of bootstrap method to derive 95% confidence interval. Logistic model is shown in blue. Gray lines represent 10,000 bootstraps of NHP dataset, to derive the 95% confidence bands (blue dashed lines) as the 250th and 9750th values of the ranked bootstraps above each ELISA value. The human EBOV GP-binding antibody data are bootstrapped 10,000 times on each of the NHP iterations to derive a 95% CI for survival probability. **b** Immunobridging of human GP-binding antibody levels from two phase I studies^23,25^ using the logistic model based on GP-binding antibody levels and survival in NHP (**a**). Green line is logistic regression model based on Ad26.ZEBOV, MVA-BN-Filo regimen with an 8-week interval. Individual human GP-binding antibody levels and the associated survival probability based on the logistic model in NHP are indicated by blue lines. The point estimate for mean predicted survival probability (magenta solid line) is the average of the individual survival probabilities. The human EBOV GP-binding antibody data (magenta open circles, *x*-axis) are bootstrapped 10,000 times on each of the NHP iterations (**b**) to derive a 95% confidence interval (CI) for mean predicted survival probability (magenta dashed lines). Survival probabilities for the main logistic model (green closed circles, *y*-axis) are shown. EU ELISA units.

The GP-binding antibody ELISA was validated for human and NHP serum, and parallel dilution curves were demonstrated for human and NHP samples (Supplementary Fig. 4), indicating that human and NHP EBOV GP-binding antibody levels can be directly compared in this assay. Figure 5a shows the logistic model for GP-binding antibodies and challenge outcome in NHP, including the bootstrap derived 95% confidence interval (CI; see also caption to Fig. 5 and “Statistical methods”). Human GP-binding antibody levels were associated with a 47.8% (95% CI: 24.1–66.3%) mean predicted survival probability (Fig. 5b). Based on the 100% mortality and extremely rapid disease progression in NHP relative to human EVD, this calculated mean survival probability demonstrates that the vaccine will have a protective effect in people, rather than being an estimate of human efficacy.

### Additional evidence for clinical benefit

We further explored the impact of vaccination on EVD in NHP by analyzing additional clinical benefits from all vaccine regimens combined, such as time to death post symptom onset and post challenge and reduction of viral load. It should be noted that the group of NHP immunized with the Ad26.ZEBOV, MVA-BN-Filo regimen in the 8-week interval comprises large numbers of NHP receiving vaccine doses well below clinical doses, where the degree of observable benefit may be lower.

Attenuated disease and delayed disease progression post symptom onset would potentially translate into an increased time window for intervention in humans, such as provision of supportive care. Overall, 11 out of 110 (10%) vaccinated NHP did not experience any EVD-specific symptoms based on recorded clinical scores (Supplementary Table 5). For NHP experiencing symptoms, time to death post symptom onset was calculated. Vaccinated NHP had extended survival post symptom onset relative to negative controls (*p* < 0.0001 log-rank test) (Supplementary Fig. 5a). Increased overall time to death post challenge (Supplementary Fig. 5b) was also observed for vaccinated, relative to unvaccinated, NHP (*p* < 0.0001, log-rank test). In nonsurvivors, GP-binding antibody titers correlated with prolonged time to death post symptom onset (Supplementary Fig. 5c) and overall time to death post challenge (Supplementary Fig. 5d), with Kendall correlations of 0.36 and 0.43, for survival post symptom onset and survival post challenge, respectively.

When exploring the impact of vaccination on EVD in NHP, a subset of vaccinated NHP did not exhibit symptoms or viremia (Supplementary Table 5). In total, 35 out of 61 (57%) surviving NHP did not have detectable serum viral load at any timepoint, as measured by qRT-PCR (Supplementary Table 5). Survival significantly correlated with absence of detectable EBOV RNA as determined by qRT-PCR and absence of observable clinical signs of EVD (*p* < 0.0001 and *p* = 0.017, respectively, Fisher’s exact test). Also, in vaccinated NHP, a logistic regression model showed a significant effect of day 5/6 viral load on survival (*p* value for the effect of viral load < 0.0001) (Supplementary Fig. 6a) and had a similar specificity and sensitivity for predicting survival outcome (ROC AUC = 0.93) relative to the logistic model based on GP-binding antibodies. Viral load in NHP showed a strong inverse relationship with GP-binding antibody level (Supplementary Fig. 6b).

Increasing GP-binding antibody levels were associated with attenuated disease progression (Supplementary Fig. 5) and reduced viral load (Supplementary Fig. 6), providing indications of additional vaccine benefit.

## Discussion

We explored whether immune markers that correlate with protection in an EBOV NHP-challenge model can be used to infer the protective effect of the same vaccine in humans. We showed that Ad26.ZEBOV, MVA-BN-Filo vaccine regimen-elicited GP-binding and neutralizing antibodies are strongly correlated with survival in the IM EBOV NHP-challenge model, which is considered the most relevant disease model for human EVD. At the selected challenge dose of 100 pfu the model is stringent, with 100% mortality and extremely rapid disease progression, and is considered an acceptable model of human EVD by regulatory authorities. GP-binding antibodies were selected as the immunological marker from which to infer the vaccine protective effect in humans, i.e., immunobridging, based on human immunogenicity data obtained in the same assay. The outcome of this exploratory immunobridging analysis indicates that it is highly likely that the immune response elicited by the Ad26.ZEBOV, MVA-BN-Filo vaccine regimen can provide protection against EVD in humans, though there is no straightforward translation to human efficacy based on the stringency of the NHP model.

There are some contradictory data with regards to the role of EBOV GP-binding antibodies in protection against EVD. While passive transfer of hyperimmune serum or monoclonal antibody cocktails demonstrated that protection against lethal EBOV infection is possible based on humoral immunity alone^28–30^, a large-scale trial using convalescent human plasma did not reveal a significant benefit^31^, though the latter could be confounded by the timing and required amount of the convalescent plasma. In NHP, the level of total EBOV GP antibodies measured by ELISA has been correlated with protection for multiple vaccine candidates^32,33^. A recent study where humoral and cellular immune responses were analyzed from different challenge experiments and across two vaccine platforms (vesicular stomatitis virus [VSV] based and Ad based) suggested that the humoral immune response, specifically the total GP-binding antibody response, plays a role in protection against EBOV challenge^22^. Different mechanisms of protection have been proposed for these two vaccine platforms. Sullivan et al. demonstrated that protection from EVD and death after EBOV challenge was abrogated in most Ad5 GP-vaccinated animals after CD8+ T-cell depletion^34^, while Marzi et al. reported the critical role of antibodies in rVSV-mediated protection, and a limited role of CD8+ T cells^32,35^. Though EBOV GP-binding antibodies may be mechanistically involved in protection, this is not a prerequisite for immunobridging based on GP-binding antibody levels.

Protection against EVD is likely to be multifactorial, whereas mechanistic studies can only probe limited immune effector mechanisms at the same time. For example, the potential contribution of the memory B cell response to protection is not captured in a passive antibody transfer study. Therefore, we used statistical modeling to assess the contribution of different immune markers to protection in a stringent NHP model of lethal EVD. We showed that protection against EBOV challenge in NHP by the Ad26.ZEBOV, MVA-BN-Filo vaccine regimen can be predicted to a high degree based on vaccine-elicited GP-binding antibody responses alone. GP-binding antibody concentrations appear to reflect EBOV neutralizing antibody responses (Fig. 3d and Supplementary Fig. 3), though it should not be assumed that all binding antibodies are also neutralizing. Either virus neutralization is an important mechanism of protection, or both binding and neutralizing antibody responses correlate with additional mechanisms involved in protection. For instance, the magnitude of T-cell responses elicited by the vaccine was also statistically correlated with protection from fatal outcome in the EBOV NHP-challenge model (Fig. 3c) but did not substantially improve the discriminatory capacity of the logistic model based on GP-binding antibodies. In fact, a logistic regression model based on GP-binding antibodies alone does not appear to require additional independent explanatory variables (Supplementary Tables 1 and 2), as indicated by the high ROC AUC (0.986) and CoD (0.78). This finding suggests that GP-binding antibodies could be mechanistically involved in protection and/or act as a surrogate of the actual protective effector mechanism(s)^36,37^.

There is no straightforward translation from vaccine efficacy in NHP to the protective effect of the vaccine in humans because of the extremely rapid disease progression in NHP, which is even faster than human needlestick infection (Fig. 1a)^38^. This could represent an intrinsic sensitivity in NHP, where doses as low as 0.01 pfu were reported to be lethal^39^, though this may depend on characteristics of the challenge material. In the challenge model used here, a dose of 100 pfu represents at least a 200-fold lethal dose, because a dose of 0.5 pfu is fully lethal (Supplementary Fig. 1). In addition, NHP are moribund within ~7 days, which does not allow for a contribution of the anamnestic response to protection, while it is anticipated that the disease course of EVD in people is slow enough to allow for a contribution of the vaccine anamnestic response to a protective effect^40^. Thus, the calculated mean survival probability based on immunobridging demonstrates that the vaccine is protective in people, rather than being an estimate of human efficacy, due to the stringency of the NHP model. Thus, assessing the durability of the vaccine protective effect would rely on an NHP model where the disease course is more reflective of human disease. In the absence of evidence for durability of protection, a booster vaccination is currently recommended as a precautionary measure upon imminent risk of exposure to maximize the protective effect^41,42^.

The high virulence of IM challenge with early passage EBOV Kikwit in NHP provides confidence that the clinical benefit inferred represents a conservative estimate of the vaccination benefit in humans. In addition to a survival benefit, we additionally explored the impact of vaccination on EVD in NHP on other aspects of potential clinical benefit, showing attenuated disease and delayed progression and reduced viral load in NHP. While the increase in survival time is limited, it represents approximately a 50% increase in survival time after onset of symptoms. This attenuation may translate into clinical benefit by augmenting the window for supportive care in humans. Viral load in NHP was correlated with survival outcome, which is also observed for natural EBOV infection in humans^13,43^.

Only one EBOV vaccine has thus far been evaluated in a field efficacy study. rVSV has demonstrated >90% efficacy using a ring vaccination setting in humans^44,45^, and high efficacy in NHP-challenge studies^32^. To our knowledge, this vaccine has not been evaluated using the same immunobridging approach described herein, taking into account the potential difference in immunogenicity of the vaccines between NHP and humans. Depending on the correlates observed, bridging antibody responses induced by Ad26.ZEBOV, MVA-BN-Filo regimen to the responses induced by rVSV vaccination^32^ could potentially allow the calibration of the predictive value of the animal model compared with natural exposure in humans. This is important to facilitate assessment of other EBOV vaccines that induce similar or higher levels of GP-binding antibody titers than rVSV as observed with Ad26.ZEBOV, MVA-BN-Filo^24^.

The anthrax vaccine BioThrax^®^ was recently licensed for an indication of postexposure prophylaxis, based on immunobridging^7^. Immunobridging methodology could also be valuable prior to the generation of effectiveness data, such as during the current COVID-19 pandemic. For instance, immunobridging of human immunogenicity to animal model effectiveness could be used to support emergency use authorization of COVID-19 vaccines. The availability of a suitable disease model currently imposes some uncertainty on immunobridging for COVID-19 vaccines, though human efficacy trials could serve to validate the predictive capacity of one or more animal models. Alternatively, bridging between immunogenicity of two vaccines in humans could be considered, once effectiveness of one candidate has been demonstrated. In the latter situation, it would be important that the selected immunological marker correlates with protection in a relevant SARS-CoV-2 animal model, and the mechanism of action is conserved between the vaccine candidates.

In summary, we showed that the Ad26.ZEBOV, MVA-BN-Filo regimen completely protected NHP from EBOV challenge and that EBOV GP-binding antibodies can be used to bridge protective efficacy in NHP to a predicted protective effect in humans. Using such a NHP logistic regression model, human immunogenicity data from completed and currently ongoing phase II/III clinical studies will be analyzed against a prespecified success criterion to demonstrate likelihood of protection against EVD of the Ad26.ZEBOV, MVA-BN-Filo vaccine regimen in humans.

## Methods

### Ethics statement

All animal research protocols were approved by either the Texas Biomedical Research Institute (TBRI, San Antonio, TX) or the United States Army Medical Research Institute for Infectious Disease (USAMRIID) Institutional Animal Care and Use Committee in compliance with the Animal Welfare Act, Public Health Service Policy on humane care and use of laboratory animals, and other federal statutes and regulations relating to animals and experiments involving animals. Vaccination phases were conducted at Bioqual (Rockville, MD), TBRI, and Battelle Biomedical Research Institute (BBRC, West Jefferson, OH). Challenge phases were conducted at TBRI or USAMRIID, both Association for the Assessment and Accreditation of Laboratory Animal Care International accredited facilities. EBOV challenge models using several different species of NHP have been described. Cynomolgus monkeys (*Macaca fascicularis*) were selected as these are most frequently used for vaccine studies, while rhesus monkeys (*Macaca mulatta*) are typically used for therapeutic studies. EVD in cynomolgus monkeys appears to have slightly more rapid progression than in rhesus monkeys^46^. Cynomolgus monkeys will be referred to as NHP, except when the species name is required.

Adult cynomolgus monkeys of Mauritian, Vietnamese, and Chinese origin were used in the studies with ~50% males and females. There was no obvious contribution of macaque origin to either vaccine immunogenicity, or challenge outcome, though this was not formally tested. Each cage had a floor area of 0.4–0.66 m^2^ and a height of 76 cm. During the course of the study, animals were provided structural (perch), inanimate (manipulable toys), and food enrichment. Food enrichment was provided on 5–7 days per week and consisted of portions of fruits and vegetables. Euthanasia was performed in accordance with the recommended method of the Panel on Euthanasia of the American Veterinary Medical Association. Animals were sedated prior to administration of an overdose of pentobarbital sodium via the intracardiac route.

Vaccinations were given at the indicated doses and vaccine composition in the quadriceps femoris, or subcutaneously for MVA-BN-Filo in one study, as a single injection with a volume of 0.5–1.0 mL. Empty vector and vector with an irrelevant insert or saline were used as negative controls. Dosing interval is indicated in the text as either 4, 6, or 8 weeks apart. Immunizations and blood draws were performed under ketamine anesthesia.

### Filovirus challenge material and monitoring

NHP studies described herein were all conducted with well-characterized early passage (P3) EBOV Kikwit material at a target dose of 100 pfu^47^. It is known that modification of experimental conditions (such as NHP species, route and dose of infection, as well as the passage number of the challenge material) can influence the course and outcome of EVD in NHP. Among these, the choice of challenge material is of crucial importance. The difference in virulence among EBOV challenge materials has been highlighted in recent publications^39,48^. Of note, subtle changes in challenge material can have a significant impact on virulence, with one still being 100% lethal at a very low inoculum dose (0.01 pfu) while 0% lethality is observed for another^39^. While the exact mechanisms implicated in these differences are not fully understood, their impact on the assessment of vaccine efficacy and, hence, on the identification of correlate(s) of protection and/or the determination of an immunological threshold associated with protection should not be underestimated. There has been a recent effort towards standardization of NHP animal models, and the current guidance by the FANG is to use an early passage of an EBOV isolate from a lethal human case, of which the identity to the human isolate has been confirmed by sequencing^49^. This challenge material maintains characteristics associated with wild-type virus (editing site of mainly 7 U residues, high particle-to-pfu ratio) and is linked to high virulence in animal models^48^. The stock used in our studies has these characteristics and was shown to be fully lethal, down to a dose of 0.5 pfu (data on file).

Viral challenges were performed with FANG approved stocks originating from lethal human infections^50^. Challenge stocks were tested to be of identical sequence to wild-type viruses by deep sequencing and were shown to be endotoxin free. All EBOV studies originated from a highly lethal Kikwit-9510621 stock, as shown in previous studies, originating from an outbreak in 1995^13,43^.

NHP were acclimatized to the BSL-4 laboratory for about 1 week. Subsequently, animals were challenged with 100 pfu EBOV Kikwit as a single IM injection in 0.5 mL volume. Animals were monitored at least twice daily after challenge and more frequently when clinical signs became apparent. A clinical scoring system was used to monitor clinical signs of disease according to an Institutional Animal Care Use Committee approved scoring sheet. Hematology and clinical chemistry parameters were recorded using either a VetScan HM2 Analyzer (Abaxis Inc.) or a COULTER Ac.T 5diff AL (Beckman Coulter Inc.). Clinical chemistry parameters were measured in serum using a VetScan analyzer or Piccolo Xpress (both Abaxis Inc.). Partial thromboplastin time (PTT) and activated PTT were measured in a Coag DX analyzer (IDEXX Laboratories Inc.). Petechial rash was recorded on clinical observation sheets at least twice daily by staff blinded to study treatment.

At TBRI, a score was assigned for general appearance, skin and fur, nose/mouth/eyes/head, respiration, feces and urine, food intake, petechiae, temperature, and locomotor activity. These scores were recorded on a daily observation sheet. A score of 8 triggered the assessment of secondary criteria to assess moribundity. At a score of ≥15, animals were euthanized by trained and experienced personnel.

At USAMRIID all animals were monitored daily and scored for disease progression. The scoring changes measured from baseline included posture/activity level, attitude/behavior, fruit/vegetable intake, respiration, and disease manifestations such as visible rash, hemorrhage and ecchymosis. A score of 3 triggered the assessment of secondary criteria to assess moribundity. A score of 4 indicated that an animal met the primary criteria for euthanasia.

A baseline score of 1 is occasionally observed at TBRI before challenge. Therefore, a clinical score ≥ 2 was used as symptom onset for studies conducted at TBRI and a clinical score ≥ 1 was used as symptom onset for USAMRIID studies.

### Vaccines and administration

Ad26.ZEBOV (Janssen Vaccines and Prevention) is a monovalent, recombinant, replication-incompetent, Ad26-vectored vaccine encoding the EBOV Mayinga GP. Ad26.Filo is composed of three Ad26-vectored vaccines encoding the EBOV Mayinga variant GP, the Sudan Gulu GP, or the Marburg Angola GP. MVA-BN-Filo (Bavarian Nordic) is a recombinant, modified vaccinia Ankara–vectored vaccine, nonreplicating in human cells, encoding the EBOV Mayinga, Sudan Gulu, Marburg Musoke GPs, and the nucleoprotein of the Tai Forest virus.

All vaccinations were given IM, except for one study where MVA-BN-Filo was given subcutaneously.

### qRT-PCR

For serum viral load qRT-PCR assays, serum was added to three volumes of TRIzol LS for inactivation, or using RNAbee (Tel-Test, Friendswood, TX, USA), followed by phase separation. RNA extraction of the aqueous phase was performed with a QIAamp Viral RNA Mini Kit. The RT-PCR reaction used SuperScript II One-Step RT-PCR System or RNA UltraSense One-Step qRT-PCR System (Invitrogen, Carlsbad, CA, USA). The genomic equivalents (GE) per mL were calculated using a standard curve of synthetic RNA of known concentration. For correlation of EBOV GP antibody concentration prior to challenge and viral load and of viral load and survival, either day 5 or 6 viral load was used depending on the sampling timepoint of the study.

### FANG EBOV GP ELISA

The EBOV GP FANG ELISA for both human and NHP serum was performed at BBRC (OH, USA). The method was described previously^51^.

### EBOV GP psVNA

Pseudovirus preparations were generated by cotransfection of human embryonic kidney (HEK) 293 cell cultures with a replication defective retroviral vector containing a luciferase gene along with an expression vector containing EBOV Makona GP sequence. Pseudovirus stocks were generated and characterized for suitability to assess EBOV-specific neutralization. Pseudoviruses were incubated with serial dilutions of serum samples and used to infect HEK293 cell cultures. Each serum sample was serially diluted ten times (fourfold), starting from a dilution of 1:40. The ability of serum to neutralize EBOV pseudovirus infectivity was assessed by measuring luciferase activity ~72 h postviral inoculation versus a control infection using a murine leukemia virus envelope (aMLV) pseudotyped virus. Neutralization titers were expressed as the reciprocal of the serum dilution that inhibited the virus infection by 50%.

### IFNγ ELISpot

Peripheral blood mononuclear cells (PBMCs) were isolated by density gradient from blood anticoagulated with EDTA and the cell concentration was adjusted to 2 × 10^6^ viable cells/mL in Roswell Park Memorial Institute (RPMI) medium supplemented with 10% fetal bovine serum (FBS), then cells rested for 1 h at 37 °C, 5% CO_2_. Polyvinylidene fluoride (PVDF) 96-well flat bottom plates precoated with the anti-monkey IFN-γ antibody were washed four times with sterile PBS (200 µL/well) and blocked with 200 µL/well of RPMI-10 (RPMI medium supplemented with 10% FBS) for 1 h at 37 °C. Then, the blocking buffer was removed from the plates and stimuli (50 µL/well) added to the appropriate wells. Conditions tested: two or three peptide pools covering the whole of EBOV Zaire Mayinga GP, each tested in the final concentration of 2 µg/mL, RPMI-10 supplemented with dimethyl sulfoxide (DMSO) as a negative control and anti-CD3 antibody diluted in RPMI-10 to a final concentration in the well of 1 µg/mL as a positive control. PBMCs (100 µL/well equivalent to 200,000 cells/well) were seeded in the plates with stimuli—all conditions were tested in duplicates. The plates were incubated (at 37 °C, 5% CO_2_) for 20 ± 1 h. After incubation, the cell suspension was removed, and plates washed five times with 1x PBS (200 µL/well). One hundred microliters of alkaline phosphatase conjugated anti-IFNγ antibody diluted 1:200 in PBS with 0.5% FBS was added to each well and allowed to bind for 2 h at room temperature. Plates were washed and 100 µL of NBT/BCIP-plus solution filtered through a 0.45 µm filter was added to each well to develop the spots for 15 min in the dark at room temperature. The reaction was stopped by extensive washing of both sides of the membranes with tap water. The plates were air-dried and read in AELVIS reader, spot counts were analyzed with EliExpert hardware version 2.1 and software version 6.1. Reportable values were calculated as average of the duplicate wells of spot forming units per million cells (SFU/10^6^ cells) after background subtraction based on the negative control stimulation (DMSO) wells.

### Statistical methods

All cross-study analyses described here are post hoc. Logistic models were used to explore the immune markers associated with the survival outcome. The logistic models are penalized logistic regression models using Firth’s method^51^ with the survival as binary outcome and each of the individual immune markers as single covariate. A 95% confidence band around each logistic model was also computed by bootstrapping 10,000 times the NHP dataset. The capacity of the logistic models to discriminate between survivors and nonsurvivors was assessed with AUC ROC curve and with the CoD^7^, which is defined as the difference in mean survival probability in the survivors and the nonsurvivors. Models with ELISA and one or two additional covariates were also considered and the potential improvement in discriminatory capacity assessed with the PPE^7^, which is the ratio of the CoD adjusted for the number of covariates for the model with additional covariates as compared with the model with ELISA as the only covariate. A 95% CI for the PPE was computed by bootstrapping the NHP dataset 10,000 times. Correlations were computed with Pearson’s method when there was evidence of linearity between the two variables and with Spearman’s method otherwise. Correlation was computed with Kendall’s method when there were many ties in at least one of the variables. The survival probability was estimated with Kaplan–Meier curves both for the time from challenge and the time from symptoms onset and difference in time to death between the vaccinated group and the negative control group was assessed with the log-rank test. The time to death was censored on the last day of follow-up for the surviving NHP.

For the immunobridging evaluation, the fitted logistic regression model for the NHP data was used to estimate a survival probability for a given human ELISA value detected at 3 weeks post dose 2. GP-binding antibody levels elicited by the Ad26.ZEBOV, MVA-BN-Filo regimen with an 8-week interval in humans (*n* = 29) were analyzed in two clinical trials^23,24^, using the same EBOV GP FANG ELISA. The individual human survival probabilities were averaged to calculate the mean predicted survival probability. For the mean predicted survival probability, a 95% CI was calculated using a nonparametric double-bootstrap method. The NHP and human datasets were resampled 10,000 times each with replacement and the logistic regression model was refitted for each resampled NHP dataset. Subsequently, predictions were made for the resampled clinical dataset based on this updated logistic regression curve. As a result, 10,000 mean predicted survival probabilities were obtained. The 95% CI was derived as the 250th and 9750th values when sorting the resulting mean predicted survival probabilities.

We further explored the impact of vaccination on EVD in NHP by analyzing additional clinical benefit based on EBOV GP-specific binding antibody levels from all vaccine regimens combined, such as time to death post symptom onset and post challenge and reduction of viral load. Penalized logistic models with Firth’s method^52^ were also used to assess the relationship between survival and the viral load at post challenge day 5/6 and for no detectable viral load at any timepoint during the study as binary outcome and ELISA as covariate. The discriminatory capacity of those models was assessed with the ROC AUC and the CoD as for the analyses of the immune markers.

Viral loads of NHP with no detectable viral load by qRT-PCR at post challenge day 5/6 were imputed at the lowest value observed across all studies (630 GE/mL).

The association between detectable viral load (any positive qRT-PCR) and survival and between detectable viral load and any detectable symptoms was evaluated with the Fisher’s exact test.

For all statistical tests that were performed, the *p* value was compared with an alpha level of 5% for significance.

All the analyses were performed with the software R (https://cran.r-project.org/) version 3.5.3.

### Reporting summary

Further information on research design is available in the Nature Research Reporting Summary linked to this article.

## Supplementary information

Supplementary Information

Reporting Summary

## Data Availability

Janssen has an agreement with the Yale Open Data Access (YODA) Project to serve as the independent review panel for evaluation of requests for CSRs and participant level data from investigators and physicians for scientific research that will advance medical knowledge and public health. Data will be made available following publication and approval by YODA of any formal requests with a defined analysis plan. For more information on this process or to make a request, please visit the Yoda Project site at http://yoda.yale.edu. The data sharing policy of Janssen Pharmaceutical Companies of Johnson & Johnson is available at https://www.janssen.com/clinical-trials/transparency.
