# Supplementary material for: Nonhuman primate to human immunobridging to infer the protective effect of an Ebola virus vaccine candidate
Source: NPJ Vaccines. 2020 Dec 17;5:112. doi: 10.1038/s41541-020-00261-9 (PMC7747701; doi:10.1038/s41541-020-00261-9)
Supplement: Supplementary file 1 — Supplementary Information [file 41541_2020_261_MOESM1_ESM.pdf]

Online supplementary material

Supplementary Figure 1: **IM challenge of cynomolgus macaques with 0.5 pfu EBOV Kikwit is fully lethal.** A group of 10 cynomolgus macaques was challenged intramuscularly with 0.5 pfu EBOV Kikwit (P3). Solid black lines indicate Kaplan-Meier survival curve. Dashed black lines indicate 95% CI around Kaplan-Meier curves

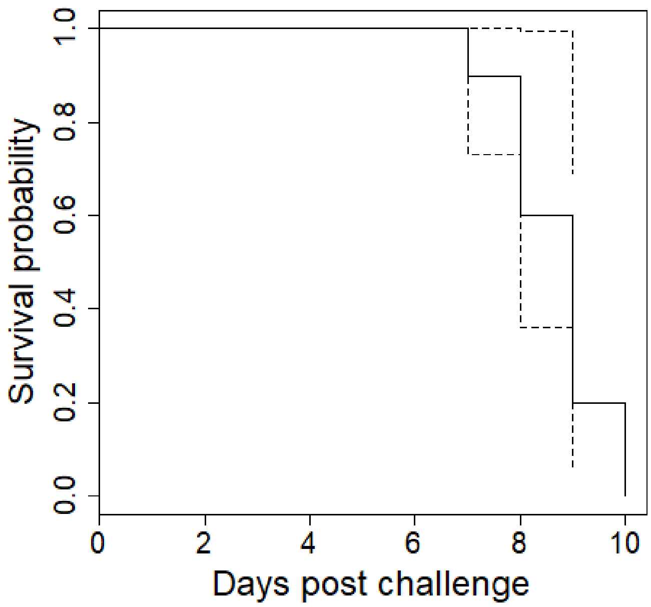

Supplementary Figure 2: Immunogenicity and protective efficacy of additional vaccine regimens tested in NHP (a) EBOV GP-binding antibody concentrations (EU/mL, $\log_{10}$ transformed) by regimen identified in panel D, as determined by FANG ELISA (BBRC); group means are indicated; dotted line is LOD. **(b)** EBOV neutralizing antibody titers (IC_{50}, $\log_{10}$ transformed) as determined by psVNA (Monogram); group means are indicated; dotted line is limit of detection (LOD). **(c)** EBOV GP-reactive IFN-γ T cells (SFU/10^6 cells, $\log_{10}$ transformed) as enumerated by IFN-γ ELISpot (TBRI); group means are indicated; dotted line is LOD. **(d)** Table identifies vaccine regimen with vaccine valency (i.e trivalent Ad26.Filo or monovalent Ad26.ZEBOV), vaccine dose, dose order and dose interval, as well as the associated survival after challenge. Data in the first column are duplicated from Fig. 2, and are included for reference.

ELISA: enzyme-linked immunosorbent assay; IFN-γ ELISpot: interferon-gamma enzyme-linked immunospot; EU: ELISA units; IC_{50}: half maximal inhibitory concentration; InfU: infectious units; psVNA: pseudovirus neutralization assay; SFU: spot forming units; PBMC: peripheral blood mononuclear cells; vp: viral particles.

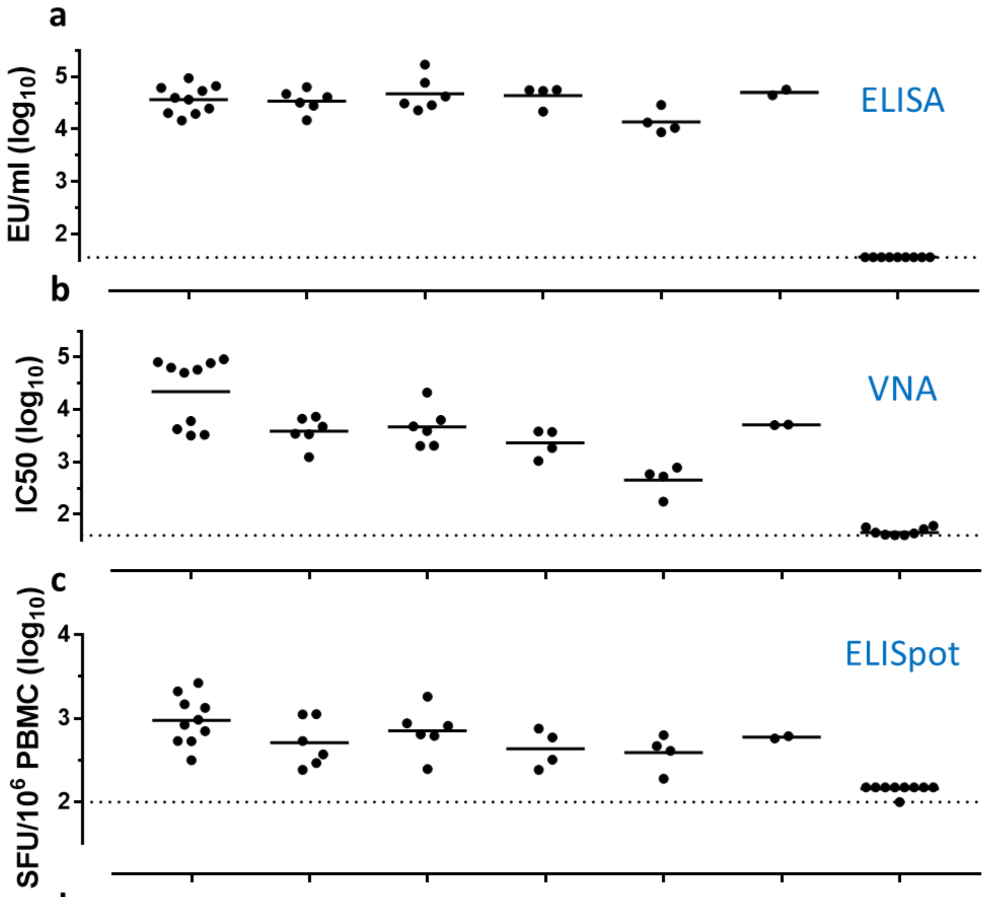

d

Dose 1	Ad26.ZEBOV $5x10^{10}$ vp	MVA-BN-Filo $5x10^{8}$ InfU	Ad26.Filo $1.2x10^{11}$ vp	Ad26.Filo $1.2x10^{11}$ vp	MVA-BN-Filo $5x10^{8}$ InfU	Ad26.Filo $1.2x10^{11}$ vp	Neg ctrl n/a
Dose 2	MVA-BN-Filo $1x10^{8}$ InfU	Ad26.Filo $1.2x10^{11}$ vp	MVA-BN-Filo $5x10^{8}$ InfU	MVA-BN-Filo $5x10^{8}$ InfU	Ad26.Filo $1.2x10^{11}$ vp	Ad26.Filo $1.2x10^{11}$ vp	Neg ctrl n/a
Dose 3						MVA-BN-Filo $5x10^{8}$ InfU	
Interval	8 weeks	8 weeks	8 weeks	4 weeks	4 weeks	4 weeks	4-8 weeks
Survival/N (%)	10/10 (100%)	5/6 (83%)	5/6 (83%)	3/4 (75%)	1/4 (25%)	2/2 (100%)	0/9 (0%)

Supplementary Figure 3: GP-binding antibody levels are reflective of EBOV neutralizing antibodies in NHP. GP-binding antibody levels (EU/mL, log_{10} transformed) elicited by A26.ZEBOV, MVA-BN-Filo vaccination with an 8-week interval were expressed as a function of EBOV neutralizing antibody titer (IC50, log_{10} transformed) and show a strong correlation.

EU: ELISA units; IC_{50}: half maximal inhibitory concentration.

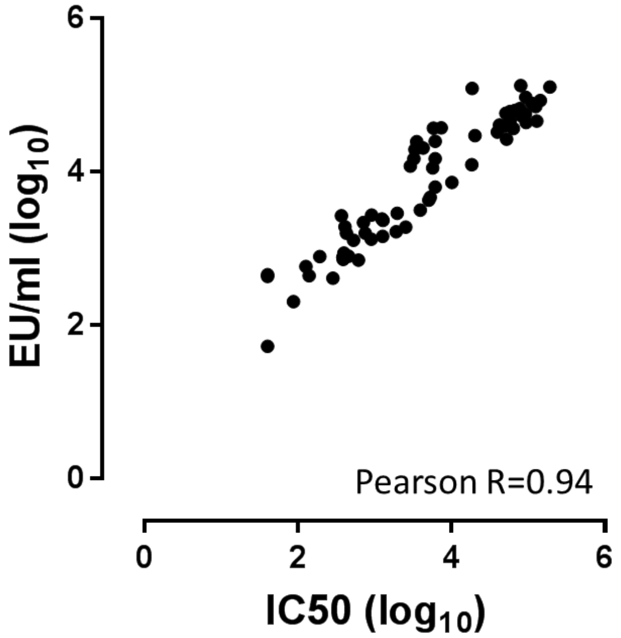

Supplementary Figure 4: Parallelism between human and NHP samples in the FANG GP-binding antibody ELISA. The parallelism assessment was based on the evaluation of the upper asymptote and slope parameters from four parameter logistic (4PL) model fit on individual sample titration curves. Left panel: Density plot of slope values for human (blue) and NHP (red) serial dilution curves in the FANG EBOV GP-binding ELISA (BBRC). Right panel: Density plot of upper asymptote values for human (blue) and NHP (red) serial dilution curves in the FANG EBOV GP-binding ELISA (BBRC).

NHP: non-human primates.

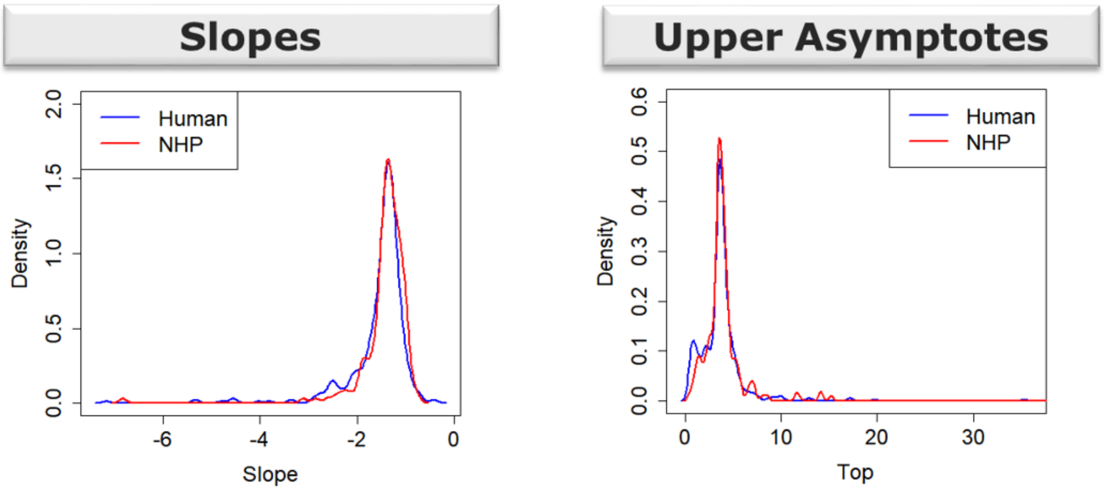

Supplementary Figure 5: Attenuated EVD progression by vaccination is associated with GP-binding antibodies. Top panels: Kaplan-Meier estimates of survival probability after symptom onset **(a)** and after challenge **(b)**. NHP that did not experience symptom onset were excluded from the analysis of survival time after symptom onset. **(a-b)** Unvaccinated NHPs (n = 13) are shown in red, vaccinated NHP (**a**, n = 99 (due to 11 NHPs without symptoms); **b**, n = 110) are shown in black. Bottom panels: Survival time after symptom onset **(c)** and after challenge (**d**) as a function of GP-binding antibody levels (EU/mL, $\log_{10}$ transformed) as determined by FANG ELISA (BBRC). Vaccinated survivors were excluded from the analysis, because their survival time is censored at the end of the follow up period. Unvaccinated controls are shown in red, vaccinated NHPs are shown in green.

EU: ELISA units.

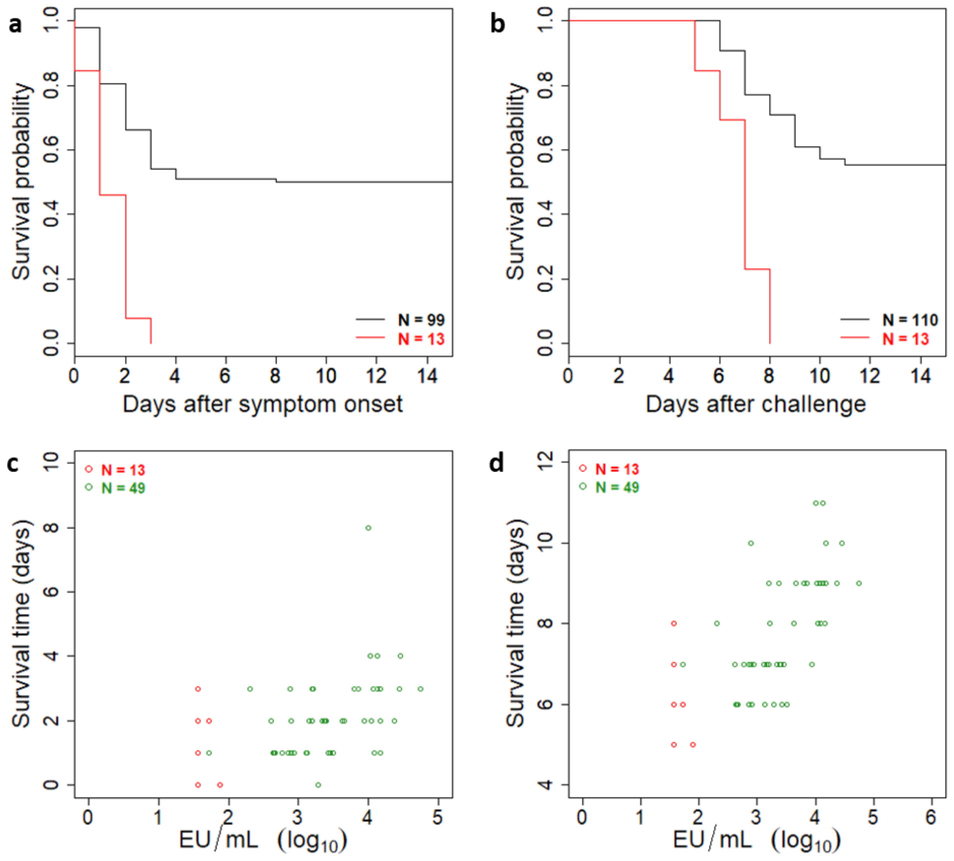

Supplementary Figure 6: Reduction of EBOV serum viral load after vaccination is related to GP-binding antibodies. (a) Logistic regression model of viral load as a predictor of survival after EBOV Kikwit challenge (100 pfu, IM) in NHP. Individual viral load levels (genome equivalents (GE)/mL, log_{10} transformed) are identified by circles and the associated survival status as a binary variable with survival as 1 and non-survival as 0. Black lines show the logistic model (solid line) and the associated 95% confidence band (dashed lines). Negative qPCR values were imputed at 630 genome equivalents (GE)/mL. **(b)** Viral load levels (GE/mL, log_{10} transformed) as a function of GP-binding antibody levels (EU/mL, log_{10} transformed) in vaccinated NHP. Negative qPCR values were imputed at 630 GE/mL. Survivors are shown in green, non-survivors are shown in red.

EU: ELISA units; GE: genome equivalents.

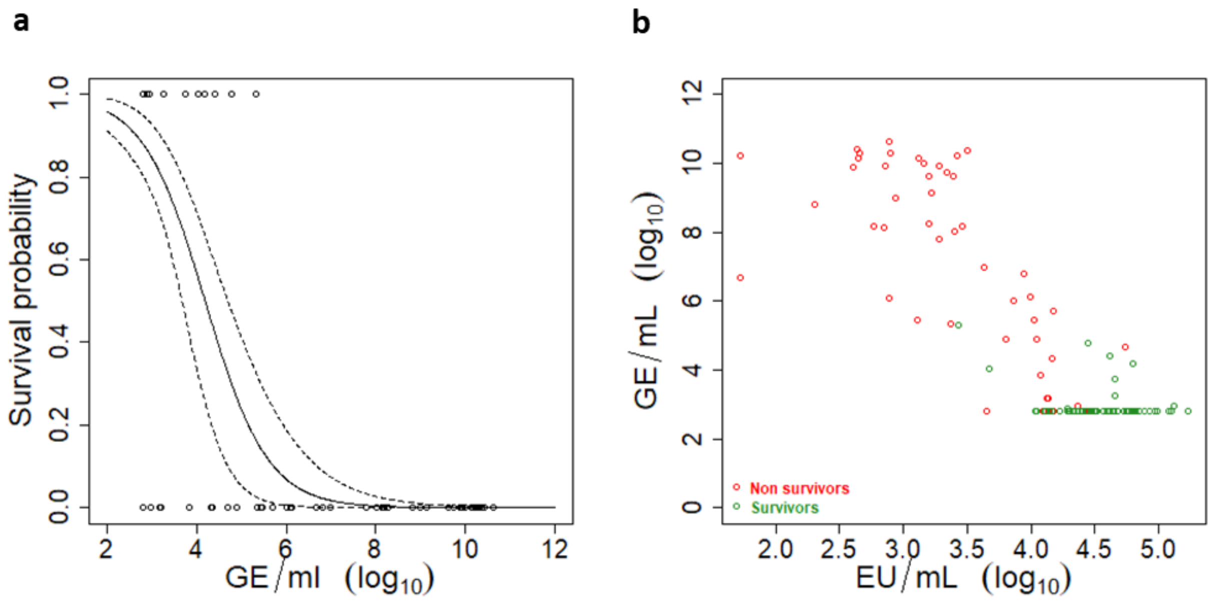

Supplementary Table 1: Summary of vaccine regimens and survival outcome across 5 NHP challenge studies.

Dose 1 (Dose, Route)	Dose 2 (Dose, Route)	Study	Dose Interval (Weeks)	Survival/N (%)
Ad26.ZEBOV ($1x10^{11}$vp, IM)	MVA-BN-Filo ($5x10^8$ Inf U, IM)	5	8	2/2 (100%)
Ad26.ZEBOV ($5x10^{10}$vp, IM)	MVA-BN-Filo ($5x10^8$ Inf U, IM)	3	8	4/4 (100%)
Ad26.ZEBOV ($5x10^{10}$vp, IM)	MVA-BN-Filo ($1x10^8$ Inf U, IM)	3	8	4/4 (100%)
Ad26.ZEBOV ($5x10^{10}$vp, IM)	MVA-BN-Filo ($1x10^8$ Inf U, IM)	5	8	6/6 (100%)
Ad26.ZEBOV ($2x10^{10}$vp, IM)	MVA-BN-Filo ($1x10^8$ Inf U, IM)	5	8	3/3 (100%)
Ad26.ZEBOV ($5x10^9$vp, IM)	MVA-BN-Filo ($1x10^8$ Inf U, IM)	5	8	4/4 (100%)
Ad26.ZEBOV ($2x10^9$vp, IM)	MVA-BN-Filo ($1x10^8$ Inf U, IM)	5	8	2/2 (100%
Ad26.Filo ($1.2x10^{11}$vp,IM)	MVA-BN-Filo ($5x10^8$ Inf U, SC)	1	8	2/2 (100%)
Ad26.Filo ($1.2x10^{11}$vp,IM)	MVA-BN-Filo ($5x10^8$ Inf U, IM)	4	8	3/4 (75%)
MVA-BN-Filo ($5x10^8$ Inf U, SC)	Ad26.Filo ($1.2x10^{11}$vp, IM)	1	8	2/2 (100%)
MVA-BN-Filo ($5x10^8$ Inf U, IM)	Ad26.Filo ($1.2x10^{11}$vp, IM)	4	8	3/4 (75%)
Ad26.ZEBOV ($5x10^{10}$vp, IM)	MVA-BN-Filo ($1x10^8$ Inf U, IM)	2	6	4/5 (80%)
Ad26.ZEBOV ($5x10^{10}$vp, IM)	MVA-BN-Filo ($1x10^8$ Inf U, IM)	2	4	2/5 (40%)
Ad26.ZEBOV ($5x10^{10}$vp, IM)	MVA-BN-Filo ($1x10^8$ Inf U, IM)	3	4	2/2 (100%)
Ad26.Filo ($1.2x10^{11}$vp,IM)	MVA-BN-Filo ($5x10^8$ Inf U, IM)	4	4	3/4 (75%)
Ad26.Filo ($1.2x10^{11}$vp,IM)	Ad26.Filo ($1.2x10^{11}$vp, IM), MVA-BN-Filo ($5x10^8$ Inf U, SC)	1	4	2/2 (100%)
MVA-BN-Filo ($1x10^8$ Inf U, IM)	Ad26.ZEBOV ($5x10^{10}$vp, IM)	3	4	2/4 (50%)
MVA-BN-Filo ($5x10^8$ Inf U, IM)	Ad26.ZEBOV ($5x10^{10}$vp, IM)	3	4	2/4 (50%)
MVA-BN-Filo ($5x10^8$ Inf U, IM)	Ad26.Filo ($1.2x10^{11}$vp, IM)	4	4	1/4 (25%)
Ad26.empty ($1.2x10^{11}$vp, IM)	MVA neg ctrl ($5x10^8$ Inf U, SC)	1	8	0/2 (0%)
Ad26.empty ($5x10^{10}$vp, IM)	MVA neg ctrl ($5x10^8$ Inf U, IM)	3	8	0/2 (0%)
Ad26.empty ($1.2x10^{11}$vp, IM)	MVA neg ctrl ($5x10^8$ Inf U, IM)	4	8	0/2 (0%)
Diluent	Diluent	5	8	0/1 (0%)
Ad26.empty ($5x10^{10}$vp,IM)	Diluent	2	4	0/2 (0%)

IM: intramuscular; Inf U: infectious units, equivalent to 50% tissue culture infective dose ($TCID_{50}$); N: number of animals; SC: subcutaneous. Study 1=C25#1, 2=12, 3=C29#1, 4=C29#2, 5=C29#8.
Differences in dose sequence, dose interval, Ad26 valency are highlighted typographically in the table. Multivalent AD26.Filo is highlighted in light grey; monovalent AD26.ZEBOV is highlighted in dark grey; a dose interval of 8 weeks is highlighted in green; a dose interval of 6 weeks is highlighted grey blue; a dose interval of 4 weeks is highlighted in light blue; the dose of MVA-BN-Filo is shown as $1x10^8$ and $5x10^8$ Inf U respectively.

Supplementary Table 2: No contribution of other covariates to discriminatory capacity of logistic model based on GP-binding antibodies (all regimens combined, n = 110).

Covariates in the Logistic Model	Adjusted CoD	PPE of the Logistic Model with ELISA Alone vs with an Additional Covariate (95% CI)	
ELISA only	0.60		
ELISA + interval between the two doses (C)	0.59	100.9%	(92.9–101.7)
ELISA + order of the two doses (F)	0.60	99.4%	(91.2–101.1)
ELISA + vaccine valency (F)	0.61	98.1%	(88.1–101.4)

CoD: coefficient of discrimination; CI: confidence interval; ELISA: enzyme-linked immunosorbent assay; PPE: percent prediction explained.

Note: ELISA data were log-transformed; F: additional covariate as factor; C: additional covariate as continuous variable

Supplementary Table 3: No contribution of vaccine doses to discriminatory capacity of logistic model based on GP-binding antibodies (Ad26.ZEBOV, MVA-BN-Filo (0, 56 days), N = 68).

Covariates in the Logistic Model	Adjusted CoD	PPE of Logistic Models with ELISA Alone vs with an Additional Covariate (95% CI)	
ELISA only	0.77		
ELISA + Ad26 dose	0.78	99.0%	(94.2–101.7)
ELISA + MVA-BN-Filo dose	0.81	95.4%	(84.8–101.9)
ELISA + Ad26 dose + MVA-BN-Filo dose	0.79	97.3%	(86.5–103.1)

CoD: coefficient of discrimination; CI: confidence interval; ELISA: enzyme-linked immunosorbent assay; PPE: percent prediction explained.

Supplementary Table 4: Summary of regimens and survival outcome across 2 NHP vaccine dose-down challenge studies.

Dose 1 (Dose, Route)	Dose 2 (Dose, Route)	Study	Dose Interval (Weeks)	Survival/N (%)
Ad26.ZEBOV (5x10^9vp, IM)	MVA-BN-Filo (1x10^8 Inf U, IM)	1	8	4/4 (100%)
Ad26.ZEBOV (5x10^9vp, IM)	MVA-BN-Filo (1x10^8 Inf U, IM)	2	8	2/2 (100%)
Ad26.ZEBOV (5x10^9vp, IM)	MVA-BN-Filo (1x10^6 Inf U, IM)	1	8	1/5 (20%)
Ad26.ZEBOV (5x10^9vp, IM)	MVA-BN-Filo (1x10^6 Inf U, IM)	2	8	0/4 (0%)
Ad26.ZEBOV (5x10^9vp, IM)	MVA-BN-Filo (1x10^5 Inf U, IM)	2	8	0/8 (0%)
Ad26.ZEBOV (5x10^9vp, IM)	MVA-BN-Filo (1x10^4 Inf U, IM)	1	8	1/5 (20%)
Ad26.ZEBOV (5x10^9vp, IM)	MVA-BN-Filo (1x10^4 Inf U, IM)	2	8	0/7 (0%)
Ad26.ZEBOV (5x10^8vp, IM)	MVA-BN-Filo (1x10^4 Inf U, IM)	1	8	0/4 (0%)
Ad26.ZEBOV (5x10^7vp, IM)	MVA-BN-Filo (1x10^4 Inf U, IM)	1	8	0/4 (0%)
Diluent	Diluent	1	8	0/2 (0%)
Diluent	Diluent	2	8	0/2 (0%)

IM: intramuscular; InfU: infectious units, equivalent to 50% tissue culture infective dose ($TCID_{50}$); N: Number of animals; SC: subcutaneous. Study 1 = TO14#1, Study 2 = TO14#2.

Monovalent ad26.ZEBOV is highlighted in dark grey; a dose interval of 8 weeks is highlighted in green; the dose of MVA-BN-Filo is shown in blue font.

Supplementary Table 5: Absence of detectable symptoms and viremia in subset of vaccinated NHP.

Outcome	All regimens (n = 110)	Ad26.ZEBOV and MVA-BN-Filo (0,56) (n = 68)	Unvaccinated controls (n = 13)
**Survival**	61/110 (55.5%)	33/68 (48.5%)	0/13 (0%)
**Without viral load**	37/110 (33.6%)	21/68 (30.9%)	0/13 (0%)
**Without symptoms**	11/110 (10%)	3/68 (4.4%)	0/13 (0%)
