# Supplementary material for: Nonhuman primate to human immunobridging to infer the protective effect of an Ebola virus vaccine candidate
Source: NPJ Vaccines. 2020 Dec 17;5:112. doi: 10.1038/s41541-020-00261-9 (PMC7747701; doi:10.1038/s41541-020-00261-9)
Supplement: Supplementary file 2 — Reporting Summary [file 41541_2020_261_MOESM2_ESM.pdf]

nature research

Corresponding author(s): Ramon Roozendaal

Last updated by author(s): 25Oct2020

Reporting Summary

Nature Research wishes to improve the reproducibility of the work that we publish. This form provides structure for consistency and transparency in reporting. For further information on Nature Research policies, see our Editorial Policies and the Editorial Policy Checklist.

Statistics

For all statistical analyses, confirm that the following items are present in the figure legend, table legend, main text, or Methods section.

n/a	Confirmed	
☐	☒	The exact sample size (*n*) for each experimental group/condition, given as a discrete number and unit of measurement
☐	☒	A statement on whether measurements were taken from distinct samples or whether the same sample was measured repeatedly
☐	☒	The statistical test(s) used AND whether they are one- or two-sided<br>*Only common tests should be described solely by name; describe more complex techniques in the Methods section.*
☐	☒	A description of all covariates tested
☐	☒	A description of any assumptions or corrections, such as tests of normality and adjustment for multiple comparisons
☐	☒	A full description of the statistical parameters including central tendency (e.g. means) or other basic estimates (e.g. regression coefficient) AND variation (e.g. standard deviation) or associated estimates of uncertainty (e.g. confidence intervals)
☒	☐	For null hypothesis testing, the test statistic (e.g. *F*, *t*, *r*) with confidence intervals, effect sizes, degrees of freedom and *P* value noted<br>*Give P values as exact values whenever suitable.*
☒	☐	For Bayesian analysis, information on the choice of priors and Markov chain Monte Carlo settings
☒	☐	For hierarchical and complex designs, identification of the appropriate level for tests and full reporting of outcomes
☐	☒	Estimates of effect sizes (e.g. Cohen's *d*, Pearson's *r*), indicating how they were calculated

Our web collection on statistics for biologists contains articles on many of the points above.

Software and code

Policy information about availability of computer code

Data collection	Microsoft Excell for Office 365 MSO (16.0.12527.20612) 32-bit, and GraphPad Prism 7.0 were used for data collection
Data analysis	All the analyses were performed with the software R (https://cran.r-project.org/) version 3.5.3.

For manuscripts utilizing custom algorithms or software that are central to the research but not yet described in published literature, software must be made available to editors and reviewers. We strongly encourage code deposition in a community repository (e.g. GitHub). See the Nature Research guidelines for submitting code & software for further information.

Data

Policy information about availability of data

All manuscripts must include a data availability statement. This statement should provide the following information, where applicable:
- Accession codes, unique identifiers, or web links for publicly available datasets
- A list of figures that have associated raw data
- A description of any restrictions on data availability

The data sharing policy of Janssen Pharmaceutical Companies of Johnson & Johnson is available at https://www.janssen.com/clinical-trials/transparency. As noted on this site, requests for access to the study data can be submitted through Yale Open Data Access (YODA) Project site at http://yoda.yale.edu.

Field-specific reporting

Please select the one below that is the best fit for your research. If you are not sure, read the appropriate sections before making your selection.

☒ Life sciences ☐ Behavioural & social sciences ☐ Ecological, evolutionary & environmental sciences

For a reference copy of the document with all sections, see nature.com/documents/nr-reporting-summary-flat.pdf

Life sciences study design

All studies must disclose on these points even when the disclosure is negative.

Sample size	Based on analysis of 5 NHP studies, it was prospectively decided to add two more NHP studies. Final analysis was performed based on 68 NHP immunized with the Ad26.ZEBOV, MVA-BN-Filo regimen in the 8-week interval. This is considered an acceptable dataset, because it balances reasonable precision with limited use of NHP.
Data exclusions	Sample volume limitations precluded analysis of 3 samples in psVNA.
Replication	psVNA and S-ELISA assays were qualified, ELISpot assay was developed. A single reportable value was generated in each of the assays.
Randomization	NHP were assigned to groups to harmonize the groups based on sex and bodyweight.
Blinding	Personnel conducting NHP studies were blinded to the group allocation during the challenge phase of the experiment

Reporting for specific materials, systems and methods

We require information from authors about some types of materials, experimental systems and methods used in many studies. Here, indicate whether each material, system or method listed is relevant to your study. If you are not sure if a list item applies to your research, read the appropriate section before selecting a response.

Materials & experimental systems

n/a	Involved in the study	
☒	☐	Antibodies
☒	☐	Eukaryotic cell lines
☒	☐	Palaeontology and archaeology
☐	☒	Animals and other organisms
☒	☐	Human research participants
☐	☒	Clinical data
☒	☐	Dual use research of concern

Methods

n/a	Involved in the study	
☒	☐	ChIP-seq
☒	☐	Flow cytometry
☒	☐	MRI-based neuroimaging

Animals and other organisms

Policy information about studies involving animals; ARRIVE guidelines recommended for reporting animal research

Laboratory animals	Crab-eating macaques (macaca fascicularis)
Wild animals	N/A
Field-collected samples	N/A
Ethics oversight	All studies were approved by local Institutional Animal Care and Use Comittees.

Note that full information on the approval of the study protocol must also be provided in the manuscript.

Clinical data

Policy information about clinical studies

All manuscripts should comply with the ICMJE guidelines for publication of clinical research and a completed CONSORT checklist must be included with all submissions.

Clinical trial registration	NCT02313077, NCT02376400
Study protocol	Anywaine, Z. et al. Safety and immunogenicity of a 2-dose heterologous vaccination regimen with Ad26.ZEBOV and MVA-BN-Filo Ebola vaccines: 12-month data from a phase 1 randomized clinical trial in Uganda and Tanzania. J. Infect. Dis. 220, 46–56 (2019). Milligan, I. D. et al. Safety and immunogenicity of novel adenovirus type 26- and modified vaccinia Ankara-vectored Ebola vaccines: a

randomized clinical trial. JAMA 315, 1610–1623 (2016).

Data collection	Anywaine, Z. et al. Safety and immunogenicity of a 2-dose heterologous vaccination regimen with Ad26.ZEBOV and MVA-BN-Filo Ebola vaccines: 12-month data from a phase 1 randomized clinical trial in Uganda and Tanzania. J. Infect. Dis. 220, 46–56 (2019). Milligan, I. D. et al. Safety and immunogenicity of novel adenovirus type 26- and modified vaccinia Ankara-vectored Ebola vaccines: a randomized clinical trial. JAMA 315, 1610–1623 (2016).
Outcomes	This manuscript contains an exploratory analysis of data generated in the studies described in the above mentioned primary publications.
